# Postpartum family planning uptake and its associated factors among postpartum women in Asosa zone, Benishangul Gumuz regional state, Ethiopia: a facility-based cross-sectional study

**DOI:** 10.1186/s40834-023-00252-w

**Published:** 2023-11-01

**Authors:** Rut Oljira, Temesgen Tilahun, Gashaw Tiruneh, Tariku Tesfaye Bekuma, Motuma Getachew, Assefa Seme, Ayantu Getahun, Lemane Dereje, Alemnesh Mosisa, Ebisa Turi

**Affiliations:** 1 Department of Public Health, Institute of Health Sciences, Wollega University, Nekemte, Ethiopia; 2 Department of Obstetrics & Gynecology, School of medicine, Institute of Health Sciences, Wollega University, Nekemte, Ethiopia; 3 Department of Public Health, College of Health sciences, Asosa University, Asossa, Ethiopia; 4 Department of Reproductive Health and Health Service Management, College of Health Sciences, Addis Ababa University, Addis Ababa, Ethiopia; 5 Department of Nursing, Institute of Health Sciences, Wollega University, Nekemte, Ethiopia

**Keywords:** Postpartum family planning, Asosa Zone, Benishangul Gumuz, Associated factors, PPFP uptake

## Abstract

**Background:**

The first twelve months after a woman has given birth is crucial for the use of contraceptives to prevent unintended pregnancy. Most women, especially in developing countries, do not realize that they are at risk for pregnancy during this period. Due to this, contraceptive use by women is ignored at this time.

**Objective:**

This study assessed the associated factors of postpartum family planning (PPFP) service uptake in the Asosa zone.

**Methods:**

A facility-based cross-sectional study was conducted among 396 postpartum women in the Asosa zone. An interviewer-administered, structured, and pre-tested questionnaire was used to collect data. Data entry and cleaning were done using Epi Info version 7.0 and analyzed using SPSS version 25 software. Multivariate logistic regression analysis was employed to identify factors associated with postpartum family planning uptake.

**Results:**

The majority of the study participants, 384 (97.2%), had heard about at least one method of family planning. Nearly two-thirds of the study participants (64.1%) had resumed sexual intercourse. Only 53.5% of the participants started using PPFP. Injectable forms (54.7%) and implants (26.4%) were the most commonly used methods. More than one-fourth (27.4%) did not use their preferred methods. Family planning use before index pregnancy (AOR = 4.8, 95% CI: 2.65, 8.82), previous use of PPFP (AOR = 2.4, 95% CI: 1.33, 4.38)] and health facility delivery (AOR = 2.8, 95% CI: 1.46, 5.49)] were significantly associated with uptake of postpartum family planning.

**Conclusion and recommendation:**

: Postpartum family planning uptake in the study area was low. Uptake of PPFP was correlated with prior family planning usage and delivery at a healthcare facility. Given these factors, we recommend all study area stakeholders to promote family planning use among women of reproductive age and to encourage deliveries at healthcare facilities. Designing a method to reach women who give birth at home for a variety of reasons is also advisable. Unavailability of different forms of FP also made the participants not use the preferred option. Therefore, we recommend the stakeholders in the study area to avail variety of FP methods.

## Introduction

Family planning (FP) is an essential component of health care provided during the antenatal and postpartum periods that can prevent maternal and childcare complications and reduce premature mortality [1]. But there are conditions in which family planning needs become unmet. Among these, the postpartum period is a critical time of high unmet needs and has the potential to reduce the risks of closely spaced pregnancies [2, 3]. Unmet need for post-partum family planning (PPFP) is defined as all sexually active and fecund women (legally married or in a consensual union) wishing to prevent unintended or closely spaced pregnancies during the first twelve months following delivery but not using any contraceptive method [4, 5]. To address these needs, postpartum family planning, which is the beginning of family planning services within the first 12 months after childbirth, has a crucial role [6].

Globally, 95% of women who are in the first 12 months of postpartum want to avoid pregnancy in the next 24 months, but 70% of them are not using contraception [7]. The most commonly used methods are relatively short-acting methods such as injections, which have high discontinuation rates [8, 9]. Also, according to Ethiopian demographic health surveillance (EDHS), 4% and 9% of pregnancies occur within less than six months and less than twelve months, respectively, after prior delivery. The unmet need in the Benishangul Gumuz region is 21.1%, and the met need is 28.5% [10], which shows a great discrepancy in the utilization of family planning.

Different studies conducted in developing countries indicated that sociodemographic characteristics, antenatal care (ANC) status, resumption of sexual activities, postnatal care (PNC), the return of menses, duration after delivery, previous history of utilization of PPFP, and place of delivery are predictors of PPFP uptake [9, 11–13].

In Ethiopia, the prevalence of contraceptive use among postpartum women varies from region to region. Most women do not start taking contraceptives at the recommended time [10]. Specifically, in this study area, the Asosa zone, utilization of family planning (FP) was low [10, 14]. Therefore, this study is aimed at assessing associated factors in the uptake of PPFP in the Asosa zone.

## Method

### Study setting and study period

A cross-sectional quantitative study design was conducted in the Benishangul Gumuz Asosa zone from September 1 to October 30, 2021. Asosa is the capital city of Benishangul Gumuz Region, Ethiopia. It is located in the Asosa Zone and is 670 km west of Addis Ababa. This town has a latitude and longitude of 10°04′ N and 34°31′ E, with an elevation of 1,570 m. The study was conducted in two hospitals (Asosa General Hospital and Menge Primary Hospital) and four health centers (Asosa Town Health Centre, Homosha Health Centre, Banbesi Health Centre, and Mender 46 Health Centre) in the zone. 49% of the population is female.

#### Study design

A cross-sectional study design was conducted.

#### Source population

All postpartum women aged between 15 and 49 years who were living in the catchment population of the study facilities.

#### Study population

Postpartum women who have given birth in the last 12 months and visiting the selected study hospitals and health centers for any maternal, neonatal, and child health (MNCH) services.

### Eligibility criteria

#### Inclusion criteria

Postpartum women who lived in the study area for at least six months and gave consent to participate in the study.

#### Exclusion criteria

Postpartum mothers who were severely sick and unable to talk were excluded from the study. Abstinence and permanent forms of contraception were not included as a form of family planning.

### Sample size determination

The sample size was determined using a single population proportion formula with the following assumptions: According to a systematic review and meta-analysis, the proportion of postpartum women who used postpartum family planning was 44% [7]. The marginal error of 5%, the design effect of 1, and the 95% confidence level were taken. After adding a 5% non-response rate, the final sample size was 402.

### Sampling techniques

All hospitals and health centers in the Asosa zone that are currently providing postpartum family planning services were considered in the study. The total list of hospitals and health centers in the zone was obtained from the zonal health department. Then, the study hospitals and health centers were selected by simple random sampling. The monthly average client load of the target age group in the MNCH department of study health facilities in the past three months prior to the study was taken from registry books (N = 804) and the respective sample size for each selected health facility was allocated proportionally to their MNCH department client flow. Finally, an eligible postpartum woman who had been selected by systematic random sampling (k = 2) was interviewed at an entry point.

### Study variables

#### Dependent variable

Postpartum family planning uptake.

#### Independent variables

socio-demographic characteristics, age, educational status, religion, ethnicity, marital status, husband education and occupation, obstetric factors, family planning-related factors, breastfeeding, facility readiness, and place of delivery.

### Data collection procedures

Data were collected by six data collectors who know Amharic and local languages. The data were collected through interviewer-administered questionnaires which were adopted from reviewed literature on postpartum family planning [8, 9, 13, 15–23]. To minimize potential bias, the authors used precise tools and data collectors who were well-trained female nurses (first degree in Bachelor of Science) who were not working in the same health facility. The overall process of data collection was supervised by one medical doctor.

### Data processing and analysis

The data were checked, entered, and cleaned using EPI-INFO version 7.0 and then exported to Statistical Package for Social Sciences (SPSS) version 25 software for analysis. Using the odds ratio (OR) with a 95% limit of the confidence interval, the association of dependent and independent variables was identified, and their degree of association was computed. Potential confounding variables were controlled by using multiple logistic regressions. Descriptive statistics like frequencies and percentages were used to describe the study population concerning dependent and independent variables. Results were presented in text, graphs, charts, and tables.

### Data quality control

The training was given to the data collectors and supervisor on the objective of the study, and how to conduct interviews. The data collection process and completeness were regularly monitored. Data collection was done in the local languages. Since this study was conducted during the initial phase of the COVID-19 pandemic, the field teams were provided the necessary personal protective equipment during the training, pretest, and actual data collection.

## Results

### Socio-demographic characteristics of participants

In this study, 396 postpartum women participated, making a response rate of 98.5%. The mean age of participants was 27.7 (SD ± 6.13). More than half, 222 (56.1%) of the participants, were aged between 20 and 29 years. Two hundred thirteen (53.8%) people resided in the urban area. Regarding the participants’ religion, more than half, 218 (55.1%), were Muslims, followed by Orthodox 108 (27.3%). Regarding the ethnicity of the study participants, Amhara, Berta, and Oromo accounted for 119 (30.1%), 113 (28.5%), and 106 (26.7%), respectively. Most (96%) of them were married. One hundred thirty four (33.8%) of the participants had no formal education (Table 1).

**Table 1:** Socio-demographic characteristics of study participants in Asosa zone, Benishangul Gumuz Regional State, 2021

Characteristics		Frequency	Percentage
**Age in years**	15 to 19	29	7.3
	20 to 24	98	24.8
	25 to 29	124	31.3
	30 to 34	93	23.5
	> 35	52	13.1
**Residence**	Urban	213	53.8
	Rural	183	46.2
**Religion**	Protestant	68	17.2
	Orthodox	108	27.3
	Muslim	218	55.1
	Others	2	0.5
**Ethnicity**	Berta	113	28.5
	Amhara	119	30.1
	Oromo	106	26.8
	Shinasha	17	4.3
	Tigre	14	3.6
	Gurage	13	3.3
	Gumuz	6	1.5
	Arab	7	1.8
	Mao	1	0.3
**Marital status**	Married	380	96
	Never married	10	2.5
	Divorced	6	1.5
**Participant education**	Cannot read and write	101	25.5
	Can read and write	33	8.3
	Grade 1to 8	88	22.2
	Grade 9 to 12	83	21
	College & above	91	23

### Reproductive history and maternal health service utilization among study participants

In this study, 270(68.2%) of the study participants were multiparous women. Three hundred seven (77.5%) of the participants attended antenatal care for the index pregnancy. The majority, 314 (79.3%), of the participants gave birth at a health facility. The mean time that the study participants stayed at health facilities after giving birth was 13.2 h (SD ± 7.44). More than half, 212(53.5%), of the study participants did not attend postnatal care. Three hundred nine (78%) of the study participants were currently breastfeeding. The result indicated that 45 (11.4%) of the participants had ever encountered an unintended pregnancy (Table 2).

**Table 2:** *Reproductive history, maternal health service utilization & Fertility intention among postpartum women attending Public health facilities* in Assossa zone, Benishangul Gumuz Regional State, 2021

*Characteristics*		*Frequency*	*Percentage*
Parity	1	126	31.8
	2 to 3	173	43.7
	≥ 4	97	24.5
ANC during the index pregnancy	Yes	307	77.5
	No	89	22.5
Place of delivery	Health facility	314	79.3
	Home	82	20.7
Postnatal care	Yes	184	46.5
	No	212	53.5
Currently breast feeding	Yes	184	46.5
	No	212	53.5
Currently living with her husband	Yes	345	90.6
	No	46	9.4
Resumed sexual intercourse	Yes	254	64.1
	No	142	35.9
Time of resuming sexual intercourse(n = 254)	6 to 12 weeks	207	81.5
	12 weeks plus one day to 24 weeks	46	18.1
	≥ 24 weeks	1	0.4
Want many more children	Yes	325	82.1
	No	71	17.9
Time to have next pregnancy	within 2 years	141	43.4
	3 to 4 years	175	53.8
	≥ 5 years	9	2.8
Planned lifetime number of children	≤ 2	30	7.6
	3 to 5	309	78
	≥ 6	57	14.4
Return of Menstruation	Yes	266	67.2
	No	130	32.8

### Fertility intention among the study participants

**Fig. 1:**
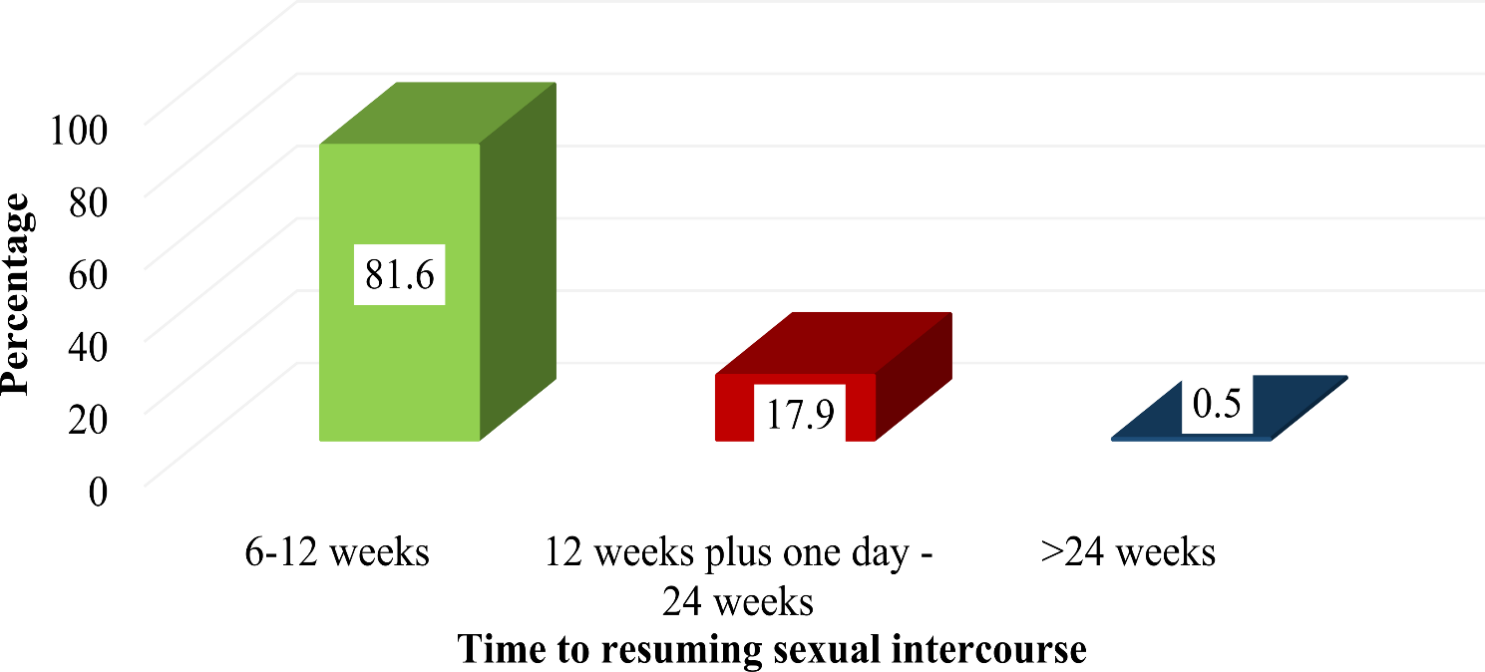
Time of resuming sexual intercourse among postpartum women at public health facilities in Asosa zone, Benishangul Gumuz Regional State, 2021

In this study, nearly two-thirds (64.1%) of the study participants had resumed sexual intercourse during the study period. Eighty-one-point-6% (81.6%) and 18% of the study participants resumed sexual intercourse between 6 and 12 weeks and 12 weeks plus one day to 24 weeks, respectively. The mean time to return to sexual intercourse was 10.4 weeks (SD + 3.66) (Fig. 1). Three hundred twenty-five (82.1%) of the study participants have expressed their desire to have many more children. More than half, 175 (54%), of the study participants want to have more children within 3 to 4 years, while 141 (43.4%) of them want it within two years. The majority, 309 (78%) of the study participants, reported that the lifetime number of children they wanted to have was 3 to 5. The mean lifetime number of children desired by the study participants was 4.2 (SD ± 1.73) (Table 3).

**Table 3:** Post-partum family planning utilization among women attending MCH clinic at Public health facilities in Asosa zone, Benishangul Gumuz Regional State, 2021

*Characteristics*		*Frequency*	*Percentage*
Previous history of using PPFP	*Yes*	184	69.2
	*No*	82	30.8
Family planning use prior to index pregnancy	*Yes*	212	53.5
	*No*	184	46.5
PPFP utilization	*Yes* *No*	212 184	53.5 46.5
Type of the method used(n = 212)	*Injectables* *Implants* *COC* *EC* *IUD*	116 56 16 13 11	54.7 26.4 7.6 6.1 5.2
Time of initiation of PPFP	*Immediately*	11	5.2
	*Within a week of delivery*	53	25
	*Between one week to 6th week*	100	47.2
	*Between 6th week to 6 months*	38	17.9
	*After 6 months*	10	4.7
Source of PPFP	*Hospital*	36	16.9
	*Health center*	156	73.6
	*Health post*	13	6.1
	*Private clinic*	5	2.4
	*Pharmacy*	2	0.9
Use of a preferred PPFP method	*Yes*	154	72.6
	*No*	58	27.4
Reason for using non-preferred methods(n = 58)	*Medical reason*	28	48.3
	*Not available*	30	51.7

### Post-partum family planning uptake among study participants

The result indicated that almost all [384 (97.2%)] of the study participants had heard about at least one method of FP. The most common form, 336 (87.5%), was the injectable contraceptive method. The participants got information about FP from health professionals in 298 (77.6%) of the study participants.

**Fig. 2:**
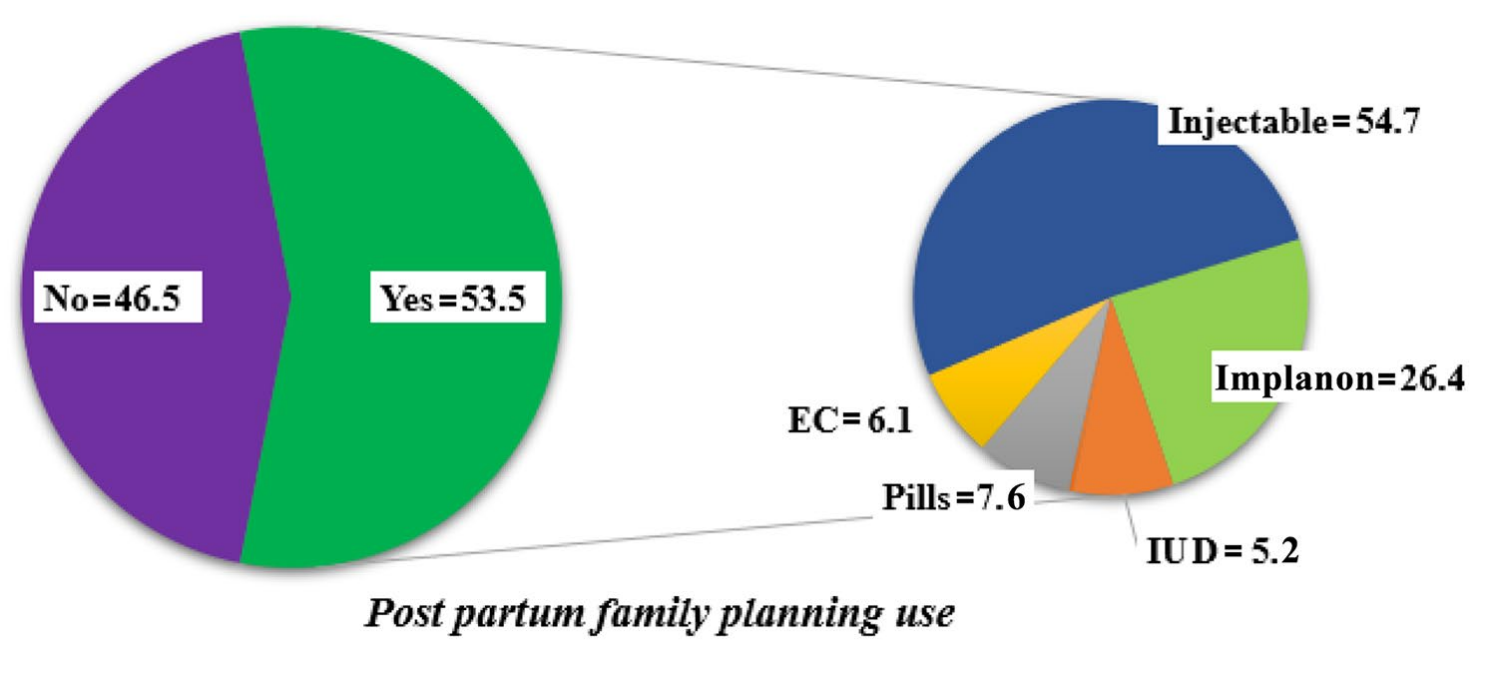
Postpartum family planning utilization among women attending MCH clinic at public health facilities in Asosa zone, Benishangul Gumuz Regional State, 2021

The majority, 293 (74%), of the study participants used any type of FP method before the index pregnancy. More than half of the study participants, 212 (53.5%), had used PPFP in the last year (during the study period). The dominant method of FP used was the injectable method, 116(54.7%), followed by Implanon, 56(26.4%). The least utilized method was the intrauterine device, 11(5.2%) (Fig. 2).

More than three-fourths, or 184 (69.2%), of the study participants had used PPFP during previous deliveries. Among those who used the postpartum family planning method, the majority, 100 (47.2%), started using it at 6 weeks. Thirty-two (15.1%) participants started using it between 6 weeks and 6 months. Only 11 (5.2%) participants started using it immediately after birth. The main sources for PPFP methods were health centers, 156 (73.6%) and hospitals 36 (16.9%). Nearly three-fourths, or 154 (72.6%), of the PPFP users got their preferred methods. The dominant reasons for preference of the method were convenience to use, 61(37.6%), and being comfortable for health, 70(43.2%). However, more than one-fourth (27.4%) did not use their preferred methods. This is due to unavailability of the preferred option, 30(51.7%) and medical reasons,28(48.3%) (Table 3).

### Associated factors to the uptake of postpartum family planning among study participants

In the multivariate analysis, some factors were significantly associated with the utilization of PPFP. These are delivery at a health facility, history of using FP before the index pregnancy, and previous history of PPFP use. On the other hand, having a history of unintended pregnancy and currently living with a husband was not significantly associated with service utilization in the study area (Table 4).

Postpartum mothers who gave birth at the health facility were more than 3 times more likely to use the PPFP service compared to those who delivered at home (AOR = 2.8, 95% CI: 1.46, 5.49). Mothers who had ever used PPFP before the index pregnancy were about 5 times more likely to uptake the service compared to those who had no history of using it (AOR = 4.8, 95% CI: 2.65, 8.82). PPFP utilization was more than 2 times higher among mothers who had used it after the preceding birth compared to their counterparts (AOR = 2.4, 95% CI: 1.33, 4.38) (Table 4).

**Table 4:** *Multivariable Logistic Regression of factors affecting PPFP service utilization among postpartum mothers attending public health facilities* in Asosa zone, Benishangul Gumuz Regional State, 2021

Variables	PPFP use		COR((95% CI))	AOR((95% CI))	P-value
	**Yes**	**No**			
**Currently living with husband**					
Yes	196 (55.1%)	160(44.9%)	1.84(0.94, 3.58)	1.98(0.91,4.29)	0.085
No	16 (40%)	24 (60%)	1	1	
**History of unintended pregnancy**					
Yes	28(62.2%)	17(37.8%)	1.50(0.79, 2.83)	0.50(0.24,1.08)	0.076
No	184 (52.4%)	167(47.6%)		1	
**FP Use before index Pregnancy**					
Yes	188 (64.2%)	105(35.8%)	5.89(3.52, 9.87)	4.83(2.645,8.817)	0.000*
No	24 (23.3%)	79 (76.7%)	1	1	
**Previous PPFP use**					
Yes	155 (57.6%)	114(42.4%)	1.67(1.09, 2.55)	2.42(1.33,4.38)	0.004*
No	57 (44.9%)	70 (55.1%)	1	1	
**Delivery Place**				1	
Health facility	192 (61.1%)	122(38.9%)	4.88 (2.81, 8.48)	2.83(1.46, 5.49)	0.002*
Home	20 (24.4%)	62 (75.6%)	1	1	

*Statistically significant association

## Discussion

Use of FP before the index pregnancy, having a history of PPFP utilization, and health facility delivery were significantly associated with PPFP uptake among postpartum women in Asosa zone, Benishangul Gumuz Regional State.

According to this study, the prevalence of PPFP utilization was 53.54%. This finding is lower when compared to the findings from the study conducted in Addis Ababa (80.3%) [16]. However, the prevalence was found to be slightly higher when compared to the results from a systematic review conducted in Ethiopia [17], a study conducted in Arba Minch [7], and Debre Berhan town [18], with a prevalence rate of 45.44%, 44%, and 41.6%, respectively. Similar studies that were conducted in sub-Saharan Africa [17], Kenya [19], and Pakistan [20] reported prevalence rates between 24.6% and 46.8%. The difference might be due to the difference in study settings (in terms of location, educational status, socioeconomic status, and reproductive health coverage) and period. In addition to this, more than half of the study participants in the current study were residents of urban areas, which could be a factor in the increased prevalence rate due to the availability of health information and increased awareness compared to those living in rural areas.

Almost all of the study participants (97.22%) had heard of at least one type of FP method. This finding is comparable with the study findings conducted in Debre Berhan [18] and Malawi [21]. This similarity of findings could be due to the increased availability of information from different health facilities and social media worldwide.

In this study, the dominant method of FP used by the study participants was found to be injectable at 54.72%. However, the study conducted in Arba Minch indicates that the majority of the study participants had used implants as a FP method, while only 13.2% had utilized injectable [7]. A study conducted in Pakistan showed that the most commonly used type of family planning method by postpartum women was condoms [20]. This discrepancy in findings might be due to differences in the availability of family planning types, study period, and levels of awareness regarding different family planning options. In this study, more than one-fourth did not use their preferred methods. This is mainly due to lack of availability of the different forms of FP. This might be because of the effects of corona virus disease as the study was conducted when the disease was a concern in the study area.

In the current study, the majority of study participants (81.6%) resumed sexual intercourse between 6 and 12 weeks. A similar study conducted in Malawi showed that 61% resumed sexual activity during this time [22]. However, a study in West Africa showed that the time to resume sexual activity is delayed beyond 1 year [23]. The difference could be explained by cultural differences among different study settings.

In this study, health facility delivery was significantly associated with the uptake of PPFP. This finding is in line with the study conducted in Uganda [9]. This could be because women who gave birth in the health facility might get appropriate postnatal care advice and counseling regarding PPFP, the return of fertility after birth, and the importance of birth spacing.

The findings of this study had clearly indicated that the use of FP before the index pregnancy and history of PPFP utilization after previous deliveries positively affected its uptake. This finding is consistent with a study conducted in Addis Ababa [16]. This could be because women who had prior experience in using FP have a better understanding of the benefits and timing of PPFP uptake.

### Limitation

This study is not without limitation. One limitation is the relatively small sample size. The other limitation is the husband, health facility and provider perspectives were not addressed.

## Conclusion and recommendation

Postpartum family planning uptake in the study area was low. Uptake of PPFP was correlated with prior FP usage and delivery at a healthcare facility. Given these factors, we recommend all study area stakeholders to promote FP use among women of reproductive age and to encourage deliveries at healthcare facilities. Designing a method to reach women who give birth at home for a variety of reasons is also advisable. Unavailability of different forms of FP also made the participants not use the preferred option. Therefore, we recommend the stakeholders in the study area to avail variety of FP methods.

## Data Availability

The data sets are available from the corresponding author on a reasonable request.

## Declarations

**Ethical Considerations**.

Ethical clearance was obtained from the Research Ethics Review Committee of Wollega University (approval number/ID = WU/IHS/043/2013. The letter was brought to the Asosa Zonal Health office. From the Zonal Health Department, a support letter was obtained and brought to the woreda Health offices and hospitals. Following an explanation of the objectives of the study, written informed consent was obtained from each study participant. The participants were assured that all information was used only for the study. Confidentiality was assured by not recording the participant’s name on the questionnaire.

## Abbreviations

ANC: antenatal care
EDHS: Ethiopian demographic health surveillance FP:family planning
IUD: intrauterine device
MNCH: maternal, neonatal, and child health
PPFP: postpartum family planning
